# Poly[[tetra­kis­(μ-2-anilinobenzoato-κ^2^ *O*:*O*′)tetra-μ_1,1,1_-azido-tetra-μ_1,1_-azido-octa­methano­lhexa­nickel(II)] methanol hexa­solvate]

**DOI:** 10.1107/S1600536811003473

**Published:** 2011-02-05

**Authors:** Yan-Ju Liu, Xian-Jiao Fu, Xi-Feng Li, Tian-Bao Qiu, Huai-Xia Yang

**Affiliations:** a Pharmacy College, Henan University of Traditional Chinese Medicine, Zhengzhou 450008, People’s Republic of China

## Abstract

The crystal structure of the title compound, [Ni_6_(C_13_H_10_NO_2_)_4_(N_3_)_8_(CH_3_OH)_8_]·6CH_3_OH, consists of a centrosymmetric hexa­nuclear [Ni^II^_6_(C_13_H_10_NO_2_)_4_(N_3_)_8_(CH_3_OH)_8_] mol­ecule and six methanol solvent mol­ecules. In the hexa­nuclear unit, the six octa­hedrally coordinated Ni^II^ atoms are linked by four μ_1,1,1_-azide and four μ_1,1_-azide bridges, forming a face-sharing Ni_6_N_8_ tetra­cubane-like unit with four missing corners. The Ni^II^ atoms are further bridged by four μ_1,2_-carboxalate groups. Neighbouring hexa­nuclear units are connected *via* N—H⋯O hydrogen-bonding inter­actions into a three-dimensional structure. Although the H atoms of the methanol OH groups could not be located, O⋯N/O contacts between 2.65 and 2.86 Å suggest that these mol­ecules participate in hydrogen bonding.

## Related literature

For background to polynuclear complexes, see: Liu *et al.* (2008 ▶). For transition metals bridged by mixed formate and azide anions, see: Liu *et al.* (2006 ▶). For related nickel(II) complexes, see: Wang *et al.* (2008 ▶).
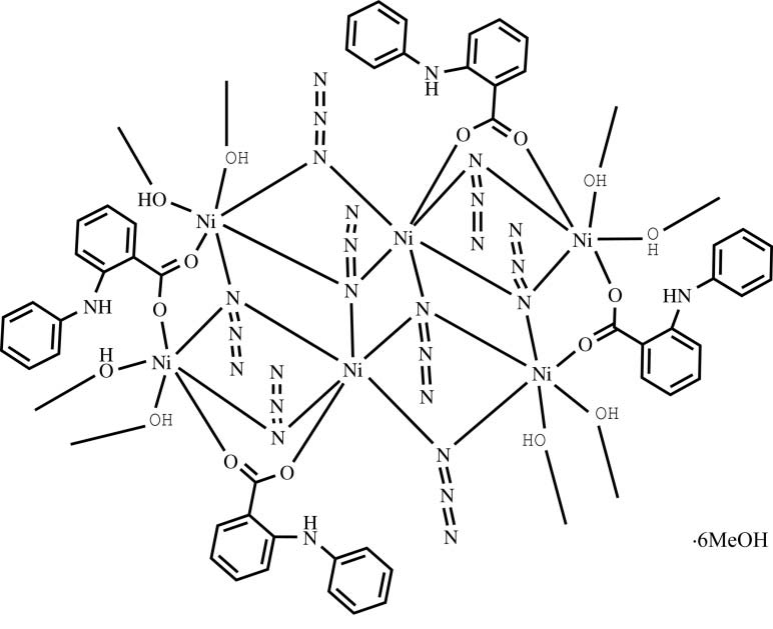


## Experimental

### 

#### Crystal data

[Ni_6_(C_13_H_10_NO_2_)_4_(N_3_)_8_(CH_4_O)_8_]·6CH_4_O
*M* *_r_* = 1985.97
Monoclinic, 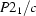

*a* = 11.8230 (1) Å
*b* = 14.6051 (2) Å
*c* = 26.3997 (4) Å
β = 105.368 (1)°
*V* = 4395.6 (1) Å^3^
*Z* = 2
Mo *K*α radiation
μ = 1.34 mm^−1^
*T* = 293 K
0.6 × 0.5 × 0.4 mm

#### Data collection

Rigaku Saturn CCD diffractometer
Absorption correction: multi-scan (*REQAB*; Jacobson, 1998 ▶) *T*_min_ = 0.461, *T*_max_ = 0.597
51748 measured reflections
7789 independent reflections
4991 reflections with *I* > 2σ(*I*)
*R*_int_ = 0.095

#### Refinement

*R*[*F* ^2^ > 2σ(*F* ^2^)] = 0.046
*wR*(*F* ^2^) = 0.127
*S* = 1.02
7789 reflections
557 parameters
1 restraint
H-atom parameters constrained
Δρ_max_ = 0.84 e Å^−3^
Δρ_min_ = −0.63 e Å^−3^

### 

Data collection: *CrystalClear* (Rigaku/MSC, 2006 ▶); cell refinement: *CrystalClear*; data reduction: *CrystalClear*; program(s) used to solve structure: *SHELXS97* (Sheldrick, 2008 ▶); program(s) used to refine structure: *SHELXL97* (Sheldrick, 2008 ▶); molecular graphics: *SHELXTL* (Sheldrick, 2008 ▶); software used to prepare material for publication: *publCIF* (Westrip, 2010 ▶).

## Supplementary Material

Crystal structure: contains datablocks I, global. DOI: 10.1107/S1600536811003473/wm2438sup1.cif

Structure factors: contains datablocks I. DOI: 10.1107/S1600536811003473/wm2438Isup2.hkl

Additional supplementary materials:  crystallographic information; 3D view; checkCIF report

## Figures and Tables

**Table 1:** Selected bond lengths (Å)

Ni1—N9	2.052 (4)
Ni1—O4	2.054 (3)
Ni1—N12^i^	2.071 (4)
Ni1—N6	2.120 (3)
Ni1—N6^i^	2.133 (3)
Ni1—N3	2.165 (3)
Ni2—O1	2.014 (3)
Ni2—O6	2.054 (3)
Ni2—O5	2.094 (3)
Ni2—N12	2.102 (4)
Ni2—N6	2.103 (3)
Ni2—N3	2.104 (3)
Ni3—O3	2.000 (3)
Ni3—O2	2.002 (3)
Ni3—O8	2.068 (3)
Ni3—O7	2.074 (3)
Ni3—N9	2.075 (4)
Ni3—N3	2.133 (4)

Symmetry code: (i) 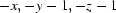.

**Table 2:** Hydrogen-bond geometry (Å, °)

*D*—H⋯*A*	*D*—H	H⋯*A*	*D*⋯*A*	*D*—H⋯*A*
N1—H1⋯O1	0.86	2.05	2.666 (5)	128
N2—H2⋯O3	0.86	2.08	2.677 (5)	126

**Fig. 1.**
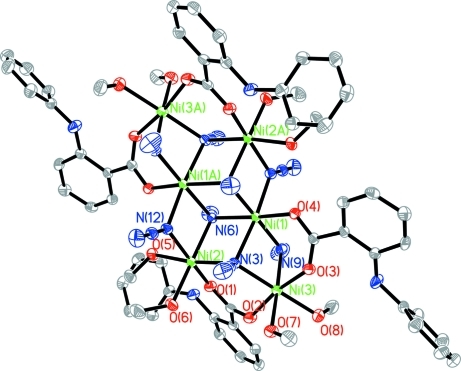
The hexanuclear unit of the title structure. The non-C atoms are labelled; all atoms are shown with displacement ellipsoids at the 30% probability level. H atoms have been omitted for clarity.

**Fig. 2.**
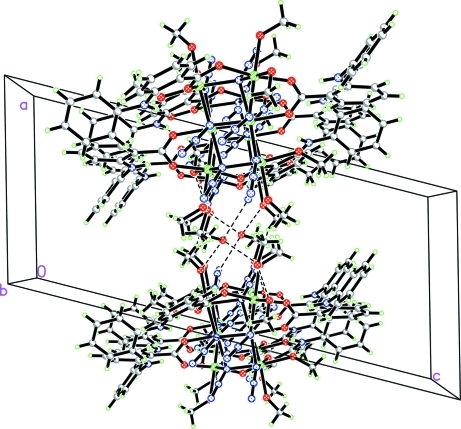
A view of the crystal packing along the b axis. Hydrogen bonding is indicated with dashed lines.

## supplementary crystallographic information

### Comment

The design and synthesis of new polynuclear metal complexes for single molecule magnets have attracted great interest in coordination chemsitry, because single molecule magnets not only show fascinating physical properties, but also have potential application in information storage and quantum computing at the molecular level (Liu *et al.*, 2008). To date, although it is difficult to predict which kind of topology and structure will lead to high-nuclearity compounds in advance, many synthetic approaches have been employed to obtain well-isolated polynuclear complexes, e.g. by using new blocking ligands with short bridges. Many single molecule magnets are based on Mn^III^, Fe^III^, and Ni^II^. In most of these compounds, magnetic exchange interactions are mainly propagated by bridging OH^-^, O*R*^-^, O^2-^, or *R*CO^2-^ groups, which often transmit antiferromagnetic interactions. An attractive strategy to facilitate the formation of ferromagnetic coupled clusters is to utilize azide and carboxalate-containing ligands simultaneously (Liu *et al.*, 2006). Herein, we report the synthesis and structure of the hexanuclear Ni(II) complex, [Ni_6_(C_13_H_10_NO_2_)_4_(N_3_)_8_(CH_3_OH)_8_]^.^6CH_3_OH.

The structure of the title compound consists of neutral hexanuclear [Ni^II^_6_(C_13_H_10_NO_2_)_4_(N_3_)_8_(CH_3_OH)_8_] molecules and six methanol solvate molecules situated between the hexanuclear units. The complete molecule has inversion symmetry. In the neutral hexanuclear unit, six octahedrally coordinated Ni^II^ atoms are linked by four µ_1,1,1_-azido and four µ_1,1_-azido bridges, forming face-sharing tetracubane units with four missing corners based on the Ni_6_N_8_ core. The Ni^II^ atoms are further bridged by four µ_1,2_-carboxalate ligands (Fig. 1). The Ni—O distances range between 2.000 (3)–2.094 (3) Å, and the Ni—N distances between 2.052 (4)–2.165 (3) Å. These bond lengths indicate that the Ni^II^ ions are in the divalent state, and are in agreement with other Ni^II^ complexes (Wang *et al.*, 2008). The hexanuclear units are connected *via* N—H···O hydrogen bonding into a three-dimensional structure (Fig. 2). The N—H···O hydrogen bonding (Table 2) is accomplished through the N atoms of 2-phenylamino-benzoate and O atoms of carboxylate groups, with the N···O distances being 2.667···2.676 Å. Although the H atoms of the methanol OH groups could not be located, short O···N/O contacts suggest that these molecules participate in hydrogen bonding between O atoms of methanol as donors and acceptors, and between O atoms of methanol and N atoms of azido bridges, with O···O distances in the range of 2.65···2.72 Å, and O···N distances in the range of 2.79···2.86 Å.

### Experimental

Under stirring, 2.0 mmol 2-phenylamino-benzoic acid, 4.0 mmol NaN_3_ were added, one after another, into a 20 ml methanol solution containing 1.0 mol Ni(ClO_4_)_2_^.^6H_2_O. The resulting solution was kept stirred for another hour, and then filtered off. The filtered solution was allowed to stand undisturbed in a sealed vessel. Crystallization took one week and gave block-shaped green crystals in a yield of 40% based on Ni(ClO_4_)_2_^.^6H_2_O. The product was washed with methanol and dried in air.

### Refinement

Hydrogen atoms bonded to C and N atoms were added geometrically and were refined using a riding model, with C—H = 0.96 Å (CH_3_), C—H = 0.93 Å (C—H) and N—H = 0.86 Å. The hydrogen atoms of the OH group of the methanol molecules could not be derived from Fourier maps and were eventually not included in the refinement.

### Crystal data

**Table:**

[Ni_6_(C_13_H_10_NO_2_)_4_(N_3_)_8_(CH_4_O)_8_]·6CH_4_O	*F*(000) = 2064
*M**_r_* = 1985.97	*D*_x_ = 1.500 Mg m^−^^3^
Monoclinic, *P*2_1_/*c*	Mo *K*α radiation, λ = 0.71073 Å
Hall symbol: -P 2ybc	Cell parameters from 62970 reflections
*a* = 11.8230 (1) Å	θ = 3.4–25.0°
*b* = 14.6051 (2) Å	µ = 1.34 mm^−^^1^
*c* = 26.3997 (4) Å	*T* = 293 K
β = 105.368 (1)°	Block, green
*V* = 4395.6 (1) Å^3^	0.6 × 0.5 × 0.4 mm
*Z* = 2	

### Data collection

**Table:**

Rigaku Saturn CCD diffractometer	7789 independent reflections
Radiation source: fine-focus sealed tube	4991 reflections with *I* > 2σ(*I*)
graphite	*R*_int_ = 0.095
Detector resolution: 0.76 pixels mm^-1^	θ_max_ = 25.1°, θ_min_ = 3.5°
ω and φ scans	*h* = −14→13
Absorption correction: multi-scan (*REQAB*; Jacobson, 1998)	*k* = −17→17
*T*_min_ = 0.461, *T*_max_ = 0.597	*l* = −31→31
51748 measured reflections	

### Refinement

**Table:**

Refinement on *F*^2^	Primary atom site location: structure-invariant direct methods
Least-squares matrix: full	Secondary atom site location: difference Fourier map
*R*[*F*^2^ > 2σ(*F*^2^)] = 0.046	Hydrogen site location: inferred from neighbouring sites
*wR*(*F*^2^) = 0.127	H-atom parameters constrained
*S* = 1.02	*w* = 1/[σ^2^(*F*_o_^2^) + (0.0602*P*)^2^ + 3.7541*P*] where *P* = (*F*_o_^2^ + 2*F*_c_^2^)/3
7789 reflections	(Δ/σ)_max_ = 0.001
557 parameters	Δρ_max_ = 0.84 e Å^−^^3^
1 restraint	Δρ_min_ = −0.63 e Å^−^^3^

### Special details

**Table:**

Geometry. All e.s.d.'s (except the e.s.d. in the dihedral angle between two l.s. planes) are estimated using the full covariance matrix. The cell e.s.d.'s are taken into account individually in the estimation of e.s.d.'s in distances, angles and torsion angles; correlations between e.s.d.'s in cell parameters are only used when they are defined by crystal symmetry. An approximate (isotropic) treatment of cell e.s.d.'s is used for estimating e.s.d.'s involving l.s. planes.
Refinement. Refinement of *F*^2^ against ALL reflections. The weighted *R*-factor *wR* and goodness of fit *S* are based on *F*^2^, conventional *R*-factors *R* are based on *F*, with *F* set to zero for negative *F*^2^. The threshold expression of *F*^2^ > σ(*F*^2^) is used only for calculating *R*-factors(gt) *etc*. and is not relevant to the choice of reflections for refinement. *R*-factors based on *F*^2^ are statistically about twice as large as those based on *F*, and *R*- factors based on ALL data will be even larger.

### Fractional atomic coordinates and isotropic or equivalent isotropic displacement parameters (Å^2^)

**Table:**

	*x*	*y*	*z*	*U*_iso_*/*U*_eq_	
Ni1	0.03270 (4)	−0.41612 (4)	−0.45991 (2)	0.02905 (16)	
Ni2	0.12781 (5)	−0.42241 (4)	−0.56219 (2)	0.03106 (16)	
Ni3	0.21549 (5)	−0.26552 (4)	−0.45100 (2)	0.03523 (17)	
O1	0.0920 (3)	−0.2905 (2)	−0.58242 (11)	0.0373 (8)	
O2	0.2149 (3)	−0.2093 (2)	−0.52023 (12)	0.0451 (8)	
O3	0.2404 (3)	−0.3091 (2)	−0.37703 (11)	0.0441 (8)	
O4	0.1078 (2)	−0.4196 (2)	−0.38032 (11)	0.0349 (7)	
O5	0.0386 (3)	−0.4489 (2)	−0.64055 (11)	0.0460 (8)	
O6	0.2908 (3)	−0.4162 (2)	−0.57582 (13)	0.0519 (9)	
O7	0.3963 (3)	−0.2654 (2)	−0.43819 (12)	0.0455 (8)	
O8	0.2376 (3)	−0.1355 (2)	−0.41891 (13)	0.0499 (9)	
N1	−0.0332 (3)	−0.2038 (3)	−0.66782 (14)	0.0429 (10)	
H1	−0.0360	−0.2487	−0.6471	0.051*	
N2	0.2946 (4)	−0.2152 (3)	−0.28674 (15)	0.0506 (11)	
H2	0.2625	−0.2080	−0.3198	0.061*	
N3	0.1967 (3)	−0.4019 (2)	−0.48097 (13)	0.0303 (8)	
N4	0.2806 (3)	−0.4517 (3)	−0.46086 (15)	0.0410 (10)	
N5	0.3583 (4)	−0.4983 (3)	−0.44295 (19)	0.0636 (13)	
N6	−0.0316 (3)	−0.4379 (2)	−0.54194 (13)	0.0261 (8)	
N7	−0.1158 (3)	−0.3975 (3)	−0.57064 (14)	0.0364 (9)	
N8	−0.1899 (4)	−0.3606 (3)	−0.59810 (19)	0.0667 (14)	
N9	0.0354 (3)	−0.2757 (3)	−0.46261 (15)	0.0368 (9)	
N10	−0.0368 (4)	−0.2270 (3)	−0.49112 (17)	0.0455 (10)	
N11	−0.1037 (5)	−0.1781 (4)	−0.5168 (2)	0.0810 (17)	
N12	0.1322 (3)	−0.5651 (2)	−0.55169 (14)	0.0347 (9)	
N13	0.2120 (4)	−0.6185 (3)	−0.54439 (15)	0.0413 (10)	
N14	0.2878 (4)	−0.6697 (3)	−0.53838 (18)	0.0626 (13)	
C1	0.1398 (4)	−0.2160 (3)	−0.56424 (17)	0.0356 (11)	
C2	0.1097 (4)	−0.1303 (3)	−0.59541 (17)	0.0349 (11)	
C3	0.1651 (4)	−0.0493 (3)	−0.57447 (18)	0.0425 (12)	
H3	0.2198	−0.0512	−0.5418	0.051*	
C4	0.1424 (5)	0.0327 (3)	−0.5999 (2)	0.0486 (13)	
H4	0.1809	0.0856	−0.5849	0.058*	
C5	0.0605 (5)	0.0357 (3)	−0.6486 (2)	0.0492 (13)	
H5	0.0447	0.0910	−0.6666	0.059*	
C6	0.0031 (4)	−0.0419 (3)	−0.67024 (18)	0.0415 (12)	
H6	−0.0535	−0.0380	−0.7022	0.050*	
C7	0.0274 (4)	−0.1275 (3)	−0.64536 (17)	0.0379 (11)	
C8	−0.0906 (4)	−0.2160 (3)	−0.72137 (17)	0.0376 (11)	
C9	−0.0428 (4)	−0.1844 (3)	−0.76057 (18)	0.0430 (12)	
H9	0.0283	−0.1532	−0.7519	0.052*	
C10	−0.1013 (5)	−0.1993 (3)	−0.81294 (18)	0.0481 (13)	
H10	−0.0695	−0.1772	−0.8392	0.058*	
C11	−0.2053 (5)	−0.2463 (3)	−0.82626 (19)	0.0479 (13)	
H11	−0.2442	−0.2558	−0.8614	0.057*	
C12	−0.2517 (4)	−0.2792 (3)	−0.7873 (2)	0.0478 (13)	
H12	−0.3221	−0.3114	−0.7960	0.057*	
C13	−0.1938 (4)	−0.2645 (3)	−0.73482 (19)	0.0419 (12)	
H13	−0.2252	−0.2876	−0.7086	0.050*	
C14	0.1895 (4)	−0.3668 (3)	−0.35553 (17)	0.0333 (11)	
C15	0.2289 (4)	−0.3742 (3)	−0.29743 (16)	0.0310 (10)	
C16	0.2167 (4)	−0.4573 (3)	−0.27362 (18)	0.0367 (11)	
H16	0.1856	−0.5072	−0.2947	0.044*	
C17	0.2492 (4)	−0.4680 (3)	−0.21992 (19)	0.0424 (12)	
H17	0.2433	−0.5247	−0.2048	0.051*	
C18	0.2909 (4)	−0.3924 (3)	−0.18871 (18)	0.0464 (13)	
H18	0.3091	−0.3979	−0.1523	0.056*	
C19	0.3054 (4)	−0.3103 (3)	−0.21058 (17)	0.0437 (12)	
H19	0.3333	−0.2607	−0.1887	0.052*	
C20	0.2793 (4)	−0.2985 (3)	−0.26533 (17)	0.0353 (11)	
C21	0.3580 (4)	−0.1407 (3)	−0.25939 (17)	0.0386 (11)	
C22	0.4703 (4)	−0.1507 (4)	−0.2265 (2)	0.0516 (13)	
H22	0.5039	−0.2087	−0.2208	0.062*	
C23	0.5326 (5)	−0.0757 (4)	−0.2021 (2)	0.0607 (15)	
H23	0.6073	−0.0836	−0.1798	0.073*	
C24	0.4856 (6)	0.0093 (4)	−0.2105 (2)	0.0637 (16)	
H24	0.5287	0.0598	−0.1948	0.076*	
C25	0.3750 (6)	0.0205 (4)	−0.2420 (2)	0.0659 (17)	
H25	0.3423	0.0788	−0.2471	0.079*	
C26	0.3105 (5)	−0.0539 (4)	−0.2668 (2)	0.0521 (14)	
H26	0.2352	−0.0452	−0.2883	0.062*	
C27	0.0697 (4)	−0.4277 (4)	−0.68775 (18)	0.0486 (13)	
H27A	0.1157	−0.3726	−0.6828	0.073*	
H27B	−0.0001	−0.4189	−0.7158	0.073*	
H27C	0.1147	−0.4772	−0.6965	0.073*	
C28	0.3410 (5)	−0.4747 (4)	−0.6070 (2)	0.0701 (17)	
H28A	0.2869	−0.5229	−0.6215	0.105*	
H28B	0.4123	−0.5007	−0.5856	0.105*	
H28C	0.3579	−0.4400	−0.6350	0.105*	
C29	0.4840 (4)	−0.2829 (4)	−0.3909 (2)	0.0619 (16)	
H29A	0.4721	−0.2436	−0.3637	0.093*	
H29B	0.5599	−0.2714	−0.3963	0.093*	
H29C	0.4793	−0.3457	−0.3809	0.093*	
C30	0.1425 (5)	−0.0758 (4)	−0.4257 (3)	0.0737 (19)	
H30A	0.1067	−0.0674	−0.4625	0.111*	
H30B	0.1694	−0.0177	−0.4099	0.111*	
H30C	0.0862	−0.1010	−0.4093	0.111*	
O9	0.4740 (3)	−0.1887 (3)	−0.02231 (14)	0.0596 (10)	
C32	0.4755 (8)	−0.2635 (6)	−0.0553 (3)	0.119 (3)	
H32A	0.3989	−0.2907	−0.0656	0.178*	
H32B	0.5313	−0.3079	−0.0369	0.178*	
H32C	0.4972	−0.2431	−0.0860	0.178*	
O10	0.6812 (4)	−0.3749 (3)	−0.08285 (19)	0.0796 (12)	
C31	0.6724 (7)	−0.3549 (5)	−0.1353 (3)	0.101 (2)	
H31A	0.6486	−0.2923	−0.1423	0.151*	
H31B	0.6153	−0.3945	−0.1574	0.151*	
H31C	0.7472	−0.3641	−0.1423	0.151*	
O11	0.6012 (4)	−0.5314 (3)	−0.05491 (19)	0.0812 (13)	
C33	0.4822 (6)	−0.5423 (5)	−0.0710 (4)	0.118 (3)	
H33A	0.4586	−0.5532	−0.1082	0.178*	
H33B	0.4446	−0.4879	−0.0631	0.178*	
H33C	0.4599	−0.5935	−0.0530	0.178*	

### Atomic displacement parameters (Å^2^)

**Table:**

	*U*^11^	*U*^22^	*U*^33^	*U*^12^	*U*^13^	*U*^23^
Ni1	0.0226 (3)	0.0415 (3)	0.0211 (3)	−0.0032 (2)	0.0023 (2)	−0.0025 (3)
Ni2	0.0255 (3)	0.0447 (4)	0.0219 (3)	−0.0044 (3)	0.0043 (2)	−0.0013 (3)
Ni3	0.0307 (3)	0.0489 (4)	0.0232 (3)	−0.0095 (3)	0.0020 (2)	−0.0018 (3)
O1	0.0361 (18)	0.0427 (19)	0.0277 (17)	−0.0090 (15)	−0.0010 (14)	0.0033 (14)
O2	0.0383 (19)	0.063 (2)	0.0272 (18)	−0.0167 (16)	−0.0030 (15)	0.0032 (15)
O3	0.0429 (19)	0.063 (2)	0.0241 (17)	−0.0212 (17)	0.0039 (15)	−0.0002 (16)
O4	0.0302 (16)	0.0460 (19)	0.0260 (16)	−0.0083 (15)	0.0033 (13)	−0.0039 (14)
O5	0.051 (2)	0.068 (2)	0.0181 (16)	−0.0173 (17)	0.0072 (15)	−0.0042 (15)
O6	0.0385 (19)	0.075 (2)	0.048 (2)	−0.0122 (18)	0.0223 (17)	−0.0148 (19)
O7	0.0293 (17)	0.067 (2)	0.0338 (19)	−0.0079 (16)	−0.0031 (15)	0.0019 (16)
O8	0.050 (2)	0.052 (2)	0.048 (2)	−0.0080 (18)	0.0133 (17)	−0.0119 (17)
N1	0.048 (2)	0.048 (2)	0.025 (2)	−0.0151 (19)	−0.0039 (18)	0.0071 (18)
N2	0.070 (3)	0.047 (3)	0.024 (2)	−0.019 (2)	−0.006 (2)	0.0023 (18)
N3	0.0187 (18)	0.046 (2)	0.025 (2)	−0.0004 (16)	0.0027 (15)	0.0002 (17)
N4	0.031 (2)	0.056 (3)	0.035 (2)	−0.010 (2)	0.0063 (19)	−0.003 (2)
N5	0.035 (3)	0.070 (3)	0.074 (3)	0.010 (2)	−0.008 (2)	0.013 (3)
N6	0.0207 (18)	0.036 (2)	0.0190 (18)	−0.0001 (16)	0.0009 (15)	0.0011 (15)
N7	0.030 (2)	0.049 (2)	0.026 (2)	−0.0042 (19)	0.0006 (19)	0.0003 (18)
N8	0.046 (3)	0.078 (3)	0.064 (3)	0.006 (3)	−0.008 (3)	0.014 (3)
N9	0.029 (2)	0.042 (2)	0.037 (2)	−0.0021 (18)	0.0050 (18)	−0.0025 (19)
N10	0.040 (2)	0.044 (3)	0.050 (3)	−0.009 (2)	0.008 (2)	−0.011 (2)
N11	0.065 (3)	0.060 (3)	0.094 (4)	0.011 (3)	−0.020 (3)	0.013 (3)
N12	0.026 (2)	0.041 (2)	0.037 (2)	0.0000 (18)	0.0088 (18)	−0.0004 (18)
N13	0.034 (2)	0.052 (3)	0.038 (2)	−0.005 (2)	0.010 (2)	−0.0036 (19)
N14	0.044 (3)	0.069 (3)	0.072 (3)	0.023 (3)	0.010 (2)	0.001 (3)
C1	0.031 (3)	0.049 (3)	0.029 (3)	−0.007 (2)	0.012 (2)	0.001 (2)
C2	0.030 (2)	0.048 (3)	0.029 (3)	−0.005 (2)	0.010 (2)	0.002 (2)
C3	0.045 (3)	0.053 (3)	0.030 (3)	−0.012 (2)	0.010 (2)	−0.004 (2)
C4	0.061 (4)	0.041 (3)	0.049 (3)	−0.011 (3)	0.023 (3)	−0.009 (3)
C5	0.063 (4)	0.043 (3)	0.051 (3)	0.000 (3)	0.030 (3)	0.002 (3)
C6	0.041 (3)	0.050 (3)	0.032 (3)	0.004 (2)	0.007 (2)	0.005 (2)
C7	0.042 (3)	0.042 (3)	0.031 (3)	−0.002 (2)	0.014 (2)	0.001 (2)
C8	0.036 (3)	0.039 (3)	0.030 (3)	−0.001 (2)	−0.004 (2)	0.003 (2)
C9	0.039 (3)	0.053 (3)	0.035 (3)	−0.006 (2)	0.006 (2)	0.005 (2)
C10	0.060 (3)	0.053 (3)	0.030 (3)	−0.001 (3)	0.008 (3)	0.003 (2)
C11	0.051 (3)	0.051 (3)	0.032 (3)	0.009 (3)	−0.006 (2)	0.001 (2)
C12	0.036 (3)	0.047 (3)	0.050 (3)	−0.001 (2)	−0.007 (2)	−0.001 (2)
C13	0.037 (3)	0.047 (3)	0.037 (3)	−0.003 (2)	0.003 (2)	0.005 (2)
C14	0.029 (2)	0.043 (3)	0.026 (2)	−0.001 (2)	0.003 (2)	−0.002 (2)
C15	0.024 (2)	0.043 (3)	0.025 (2)	−0.001 (2)	0.0047 (19)	−0.003 (2)
C16	0.030 (2)	0.042 (3)	0.038 (3)	−0.001 (2)	0.008 (2)	−0.003 (2)
C17	0.042 (3)	0.044 (3)	0.042 (3)	−0.002 (2)	0.013 (2)	0.012 (2)
C18	0.049 (3)	0.060 (3)	0.024 (3)	−0.004 (3)	−0.001 (2)	0.009 (2)
C19	0.050 (3)	0.052 (3)	0.024 (3)	−0.011 (2)	0.000 (2)	−0.009 (2)
C20	0.033 (2)	0.046 (3)	0.025 (2)	−0.006 (2)	0.003 (2)	0.001 (2)
C21	0.044 (3)	0.047 (3)	0.024 (2)	−0.009 (2)	0.007 (2)	−0.002 (2)
C22	0.045 (3)	0.055 (3)	0.052 (3)	−0.001 (3)	0.006 (3)	−0.003 (3)
C23	0.043 (3)	0.081 (5)	0.055 (4)	−0.017 (3)	0.007 (3)	−0.011 (3)
C24	0.071 (4)	0.065 (4)	0.056 (4)	−0.029 (3)	0.019 (3)	−0.018 (3)
C25	0.091 (5)	0.043 (3)	0.064 (4)	−0.001 (3)	0.021 (4)	−0.008 (3)
C26	0.053 (3)	0.053 (3)	0.046 (3)	0.001 (3)	0.006 (3)	−0.003 (3)
C27	0.052 (3)	0.066 (4)	0.029 (3)	−0.003 (3)	0.012 (2)	0.001 (2)
C28	0.045 (3)	0.095 (5)	0.077 (4)	−0.001 (3)	0.029 (3)	−0.009 (4)
C29	0.035 (3)	0.098 (5)	0.043 (3)	0.001 (3)	−0.006 (3)	0.004 (3)
C30	0.055 (4)	0.071 (4)	0.102 (5)	−0.012 (3)	0.031 (4)	−0.038 (4)
O9	0.043 (2)	0.087 (3)	0.049 (2)	0.0074 (19)	0.0117 (18)	−0.007 (2)
C32	0.113 (7)	0.132 (7)	0.111 (7)	0.041 (5)	0.030 (5)	−0.032 (6)
O10	0.067 (3)	0.081 (3)	0.087 (3)	−0.007 (2)	0.014 (2)	−0.002 (3)
C31	0.089 (6)	0.118 (6)	0.084 (6)	−0.008 (5)	0.005 (4)	0.005 (5)
O11	0.059 (3)	0.074 (3)	0.115 (4)	0.019 (2)	0.030 (3)	0.009 (3)
C33	0.070 (5)	0.079 (5)	0.202 (10)	0.013 (4)	0.028 (6)	0.012 (6)

### Geometric parameters (Å, °)

**Table:**

Ni1—N9	2.052 (4)	C9—C10	1.390 (6)
Ni1—O4	2.054 (3)	C9—H9	0.9300
Ni1—N12^i^	2.071 (4)	C10—C11	1.370 (7)
Ni1—N6	2.120 (3)	C10—H10	0.9300
Ni1—N6^i^	2.133 (3)	C11—C12	1.375 (7)
Ni1—N3	2.165 (3)	C11—H11	0.9300
Ni2—O1	2.014 (3)	C12—C13	1.389 (6)
Ni2—O6	2.054 (3)	C12—H12	0.9300
Ni2—O5	2.094 (3)	C13—H13	0.9300
Ni2—N12	2.102 (4)	C14—C15	1.484 (6)
Ni2—N6	2.103 (3)	C15—C16	1.392 (6)
Ni2—N3	2.104 (3)	C15—C20	1.424 (6)
Ni3—O3	2.000 (3)	C16—C17	1.376 (6)
Ni3—O2	2.002 (3)	C16—H16	0.9300
Ni3—O8	2.068 (3)	C17—C18	1.387 (6)
Ni3—O7	2.074 (3)	C17—H17	0.9300
Ni3—N9	2.075 (4)	C18—C19	1.362 (6)
Ni3—N3	2.133 (4)	C18—H18	0.9300
O1—C1	1.262 (5)	C19—C20	1.406 (6)
O2—C1	1.265 (5)	C19—H19	0.9300
O3—C14	1.255 (5)	C21—C26	1.379 (7)
O4—C14	1.273 (5)	C21—C22	1.388 (6)
O5—C27	1.424 (5)	C22—C23	1.380 (7)
O6—C28	1.422 (6)	C22—H22	0.9300
O7—C29	1.418 (5)	C23—C24	1.354 (8)
O8—C30	1.396 (6)	C23—H23	0.9300
N1—C7	1.371 (6)	C24—C25	1.360 (8)
N1—C8	1.408 (5)	C24—H24	0.9300
N1—H1	0.8601	C25—C26	1.387 (7)
N2—C20	1.373 (6)	C25—H25	0.9300
N2—C21	1.408 (6)	C26—H26	0.9300
N2—H2	0.8602	C27—H27A	0.9600
N3—N4	1.231 (5)	C27—H27B	0.9600
N4—N5	1.140 (5)	C27—H27C	0.9600
N6—N7	1.231 (5)	C28—H28A	0.9600
N6—Ni1^i^	2.133 (3)	C28—H28B	0.9600
N7—N8	1.116 (5)	C28—H28C	0.9600
N9—N10	1.209 (5)	C29—H29A	0.9600
N10—N11	1.145 (6)	C29—H29B	0.9600
N12—N13	1.199 (5)	C29—H29C	0.9600
N12—Ni1^i^	2.071 (4)	C30—H30A	0.9600
N13—N14	1.146 (5)	C30—H30B	0.9600
C1—C2	1.488 (6)	C30—H30C	0.9600
C2—C3	1.394 (6)	O9—C32	1.399 (7)
C2—C7	1.417 (6)	C32—H32A	0.9600
C3—C4	1.364 (7)	C32—H32B	0.9600
C3—H3	0.9300	C32—H32C	0.9600
C4—C5	1.391 (7)	O10—C31	1.391 (8)
C4—H4	0.9300	C31—H31A	0.9600
C5—C6	1.366 (7)	C31—H31B	0.9600
C5—H5	0.9300	C31—H31C	0.9600
C6—C7	1.406 (6)	O11—C33	1.367 (8)
C6—H6	0.9300	C33—H33A	0.9600
C8—C13	1.373 (6)	C33—H33B	0.9600
C8—C9	1.384 (6)	C33—H33C	0.9600
			
N9—Ni1—O4	93.08 (13)	C6—C7—C2	117.7 (4)
N9—Ni1—N12^i^	99.26 (15)	C13—C8—C9	119.3 (4)
O4—Ni1—N12^i^	90.83 (13)	C13—C8—N1	119.0 (4)
N9—Ni1—N6	96.84 (14)	C9—C8—N1	121.6 (4)
O4—Ni1—N6	169.12 (12)	C8—C9—C10	119.8 (5)
N12^i^—Ni1—N6	91.98 (13)	C8—C9—H9	120.1
N9—Ni1—N6^i^	179.03 (14)	C10—C9—H9	120.1
O4—Ni1—N6^i^	87.46 (12)	C11—C10—C9	120.7 (5)
N12^i^—Ni1—N6^i^	81.53 (14)	C11—C10—H10	119.6
N6—Ni1—N6^i^	82.56 (13)	C9—C10—H10	119.6
N9—Ni1—N3	82.65 (14)	C10—C11—C12	119.5 (5)
O4—Ni1—N3	95.22 (12)	C10—C11—H11	120.3
N12^i^—Ni1—N3	173.55 (14)	C12—C11—H11	120.3
N6—Ni1—N3	81.66 (12)	C11—C12—C13	120.2 (5)
N6^i^—Ni1—N3	96.51 (13)	C11—C12—H12	119.9
O1—Ni2—O6	93.00 (13)	C13—C12—H12	119.9
O1—Ni2—O5	84.23 (12)	C8—C13—C12	120.5 (5)
O6—Ni2—O5	94.91 (13)	C8—C13—H13	119.8
O1—Ni2—N12	168.48 (13)	C12—C13—H13	119.8
O6—Ni2—N12	94.32 (14)	O3—C14—O4	124.3 (4)
O5—Ni2—N12	86.29 (14)	O3—C14—C15	117.3 (4)
O1—Ni2—N6	91.76 (13)	O4—C14—C15	118.3 (4)
O6—Ni2—N6	174.19 (14)	C16—C15—C20	119.0 (4)
O5—Ni2—N6	88.87 (12)	C16—C15—C14	119.2 (4)
N12—Ni2—N6	81.51 (13)	C20—C15—C14	121.7 (4)
O1—Ni2—N3	97.55 (13)	C17—C16—C15	122.0 (4)
O6—Ni2—N3	92.54 (13)	C17—C16—H16	119.0
O5—Ni2—N3	172.24 (13)	C15—C16—H16	119.0
N12—Ni2—N3	91.00 (14)	C16—C17—C18	118.7 (4)
N6—Ni2—N3	83.53 (13)	C16—C17—H17	120.6
O3—Ni3—O2	170.33 (12)	C18—C17—H17	120.6
O3—Ni3—O8	85.53 (13)	C19—C18—C17	120.9 (4)
O2—Ni3—O8	88.09 (13)	C19—C18—H18	119.5
O3—Ni3—O7	87.85 (13)	C17—C18—H18	119.5
O2—Ni3—O7	84.40 (13)	C18—C19—C20	121.7 (4)
O8—Ni3—O7	85.37 (13)	C18—C19—H19	119.1
O3—Ni3—N9	90.07 (14)	C20—C19—H19	119.1
O2—Ni3—N9	98.02 (14)	N2—C20—C19	121.0 (4)
O8—Ni3—N9	98.04 (14)	N2—C20—C15	121.5 (4)
O7—Ni3—N9	175.86 (14)	C19—C20—C15	117.4 (4)
O3—Ni3—N3	92.25 (13)	C26—C21—C22	118.1 (4)
O2—Ni3—N3	93.99 (13)	C26—C21—N2	119.8 (4)
O8—Ni3—N3	177.59 (14)	C22—C21—N2	122.1 (5)
O7—Ni3—N3	93.61 (13)	C23—C22—C21	120.8 (5)
N9—Ni3—N3	82.89 (14)	C23—C22—H22	119.6
C1—O1—Ni2	133.1 (3)	C21—C22—H22	119.6
C1—O2—Ni3	129.7 (3)	C24—C23—C22	120.4 (5)
C14—O3—Ni3	133.7 (3)	C24—C23—H23	119.8
C14—O4—Ni1	125.1 (3)	C22—C23—H23	119.8
C27—O5—Ni2	130.1 (3)	C23—C24—C25	119.8 (5)
C28—O6—Ni2	129.1 (3)	C23—C24—H24	120.1
C29—O7—Ni3	128.7 (3)	C25—C24—H24	120.1
C30—O8—Ni3	120.7 (3)	C24—C25—C26	120.8 (6)
C7—N1—C8	126.5 (4)	C24—C25—H25	119.6
C7—N1—H1	116.7	C26—C25—H25	119.6
C8—N1—H1	116.8	C21—C26—C25	120.1 (5)
C20—N2—C21	125.7 (4)	C21—C26—H26	120.0
C20—N2—H2	117.1	C25—C26—H26	120.0
C21—N2—H2	117.2	O5—C27—H27A	109.5
N4—N3—Ni2	113.9 (3)	O5—C27—H27B	109.5
N4—N3—Ni3	113.6 (3)	H27A—C27—H27B	109.5
Ni2—N3—Ni3	119.03 (16)	O5—C27—H27C	109.5
N4—N3—Ni1	120.4 (3)	H27A—C27—H27C	109.5
Ni2—N3—Ni1	96.71 (13)	H27B—C27—H27C	109.5
Ni3—N3—Ni1	90.38 (13)	O6—C28—H28A	109.5
N5—N4—N3	179.0 (5)	O6—C28—H28B	109.5
N7—N6—Ni2	115.2 (3)	H28A—C28—H28B	109.5
N7—N6—Ni1	124.7 (3)	O6—C28—H28C	109.5
Ni2—N6—Ni1	98.09 (13)	H28A—C28—H28C	109.5
N7—N6—Ni1^i^	119.0 (3)	H28B—C28—H28C	109.5
Ni2—N6—Ni1^i^	97.17 (13)	O7—C29—H29A	109.5
Ni1—N6—Ni1^i^	97.44 (13)	O7—C29—H29B	109.5
N8—N7—N6	177.4 (5)	H29A—C29—H29B	109.5
N10—N9—Ni1	126.5 (3)	O7—C29—H29C	109.5
N10—N9—Ni3	125.2 (3)	H29A—C29—H29C	109.5
Ni1—N9—Ni3	95.24 (16)	H29B—C29—H29C	109.5
N11—N10—N9	177.3 (5)	O8—C30—H30A	109.5
N13—N12—Ni1^i^	128.4 (3)	O8—C30—H30B	109.5
N13—N12—Ni2	131.1 (3)	H30A—C30—H30B	109.5
Ni1^i^—N12—Ni2	99.16 (16)	O8—C30—H30C	109.5
N14—N13—N12	178.7 (5)	H30A—C30—H30C	109.5
O1—C1—O2	123.4 (4)	H30B—C30—H30C	109.5
O1—C1—C2	119.9 (4)	O9—C32—H32A	109.5
O2—C1—C2	116.7 (4)	O9—C32—H32B	109.5
C3—C2—C7	118.7 (4)	H32A—C32—H32B	109.5
C3—C2—C1	118.4 (4)	O9—C32—H32C	109.5
C7—C2—C1	122.9 (4)	H32A—C32—H32C	109.5
C4—C3—C2	122.7 (5)	H32B—C32—H32C	109.5
C4—C3—H3	118.7	O10—C31—H31A	109.5
C2—C3—H3	118.7	O10—C31—H31B	109.5
C3—C4—C5	118.7 (5)	H31A—C31—H31B	109.5
C3—C4—H4	120.6	O10—C31—H31C	109.5
C5—C4—H4	120.6	H31A—C31—H31C	109.5
C6—C5—C4	120.5 (5)	H31B—C31—H31C	109.5
C6—C5—H5	119.8	O11—C33—H33A	109.5
C4—C5—H5	119.8	O11—C33—H33B	109.5
C5—C6—C7	121.7 (5)	H33A—C33—H33B	109.5
C5—C6—H6	119.2	O11—C33—H33C	109.5
C7—C6—H6	119.2	H33A—C33—H33C	109.5
N1—C7—C6	120.1 (4)	H33B—C33—H33C	109.5
N1—C7—C2	122.0 (4)		

Symmetry codes: (i) −*x*, −*y*−1, −*z*−1.

### Hydrogen-bond geometry (Å, °)

**Table:**

*D*—H···*A*	*D*—H	H···*A*	*D*···*A*	*D*—H···*A*
N1—H1···O1	0.86	2.05	2.666 (5)	128
N2—H2···O3	0.86	2.08	2.677 (5)	126
